# Extracellular vesicles from the inflammatory microenvironment regulate the osteogenic and odontogenic differentiation of periodontal ligament stem cells by miR-758-5p/LMBR1/BMP2/4 axis

**DOI:** 10.1186/s12967-022-03412-9

**Published:** 2022-05-13

**Authors:** Chaoting Yan, Na Li, Tong Xiao, Xiaying Ye, Lin Fu, Yu Ye, Tao Xu, Jinhua Yu

**Affiliations:** 1 Key Laboratory of Oral Diseases of Jiangsu Province, Stomatological Institute, Nanjing Medical University, 136 Hanzhong Road, Nanjing, 210029 Jiangsu China; 2 Endodontic Department, School of Stomatology, Nanjing Medical University, 136 Hanzhong Road, Nanjing, 210029 Jiangsu China; 3 Department of Stomatology, The First People’s Hospital of Lianyungang, Lianyungang Clinical Medical College, Nanjing Medical University, Lianyungang, China

**Keywords:** Extracellular vesicle, Osteogenic and odontogenic differentiation, Periodontal ligament stem cell, Dental pulp stem cell, miRNA sequence

## Abstract

**Background:**

Extracellular vesicles (EVs) play a key role in constructing a microenvironment that favors the differentiation of stem cells. The present work aimed to determine the molecular mechanisms by which EV derived from inflammatory dental pulp stem cell (iDPSC-EV) influence periodontal ligament stem cells (PDLSCs) and provide a potential strategy for bone and dental pulp regeneration.

**Methods:**

The osteogenic and odontogenic differentiation was assessed by quantitative real-time polymerase chain reaction (qRT-PCR), western blot, alkaline phosphatase (ALP) activity assay, ALP staining, alizarin red S (ARS) staining, and immunofluorescence staining. To detect proliferation, the Cell Counting Kit-8 (CCK-8) assay, and flow cytometry analysis were used. EVs were isolated by the Exoperfect kit and ultrafiltration and characterized by transmission electron microscopy (TEM), nanoparticle tracking analysis (NTA), and western blot. The expression profile of miRNAs in EVs was studied using miRNA sequence and bioinformatics, and one of the upregulated miRNAs was evaluated on PDLSCs.

**Results:**

The inflammatory microenvironment stimulated osteogenic and odontogenic differentiation of DPSCs and iDPSC-EV behaved alike on PDLSCs. MiR-758-5p was upregulated in iDPSC-EV and was demonstrated to play a significant role in the osteogenic and odontogenic commitment of PDLSCs. A dual-luciferase reporter assay confirmed the binding site between miR-758-5p and limb development membrane protein 1 (*LMBR1*). The knockdown of LMBR1 also enhanced the above potential. Mechanically, bone morphogenetic protein (BMP) signaling was activated.

**Conclusions:**

EVs from the inflammatory microenvironment enhanced the osteogenic and odontogenic differentiation of PDLSCs partly by shuttering LMBR1-targeting miR-758-5p via BMP signaling.

**Supplementary Information:**

The online version contains supplementary material available at 10.1186/s12967-022-03412-9.

## Background

Oral-facial hard tissue defects induced by trauma, surgery, or congenital malformations result in functional and cosmetic issues for individuals and inflict a financial and public health cost on society [1, 2]. Dental mesenchyme stem cells, especially dental pulp stem cells (DPSCs) and periodontal ligament stem cells (PDLSCs), can seed tissue engineer regeneration [3]. They are favorable candidates in clinical application with multiple superiorities–differentiation potential, self-renewal, proliferative potential, homing, and injury repairing [4]. In recent years, extracellular vesicles (EVs) have been identified as paracrine effectors that mediate the connection between stem cells and tissue regeneration. They share their originating cells’ charters, ensuring their safety throughout the application, and helping to develop a favorable milieu for cell-free therapies [5–7]. These secretory particles can also migrate toward wounds, thus speeding up the healing process [8]. As vehicles capable of cell-to-cell communication, EVs’ cargoes varied according to their origins, cell culture methods, and isolation methods, among other factors [9, 10]. Dental pulp stem cell EV (DPSC-EV) is validated as an operational tool for bone tissue regeneration in vivo [11]. However, it can also form a dentin-pulp complex, inhibit apoptosis, and is effective in angiogenesis [5, 12, 13]. In vitro, PDLSC has the potential of odontogenic differentiation and can be triggered into osteoblast by adding EVs [14, 15]. The biological function of EVs is mainly derived from noncoding RNAs, especially micro RNAs (miRNAs), which are important participators in epigenetic regulation [16]. MiRNAs, which are non-coding RNAs of 18–30 nucleotides, contribute to expression and mediate post-transcriptional and translational mRNA levels, and have been a study focus in recent decades [17]. While the development of RNA-induced silencing complexes is a well-established concept for miRNA biogenesis and function, new hypotheses and research approaches continue to emerge [18]. The revelation that EVs assemble certain miRNA to perform specific biological functions has steadily become a focus of interest for researchers [13]. Moreover, mesenchymal stem cell (MSC) that have been functionally engineered with specific proteins do not change the protein cargoes, but the miRNA composition of EVs changes as a result of the modification [19], meaning that overexpression of miRNA in parent cells can transfer to EVs and make EVs behave alike its origin [20].

Inflammatory and non-inflammatory events are critical in determining the fate of cells during repair and regeneration in the microenvironment [21]. Repair dentine development (tertiary dentine), which results from the interaction of inflammation and host defense responses, inspired researchers to explore aberrant differentiation, regeneration and repair [22]. Inflammatory cytokines activate bone marrow mesenchymal stem cells and increase their function as osteoblasts [23]. A comparable investigation demonstrates that 48-h treatment with 10 ng/mL TNF-α increases the ability of DPSC to differentiate into osteoblasts and odontoblasts [24]. TNF-α administration at 10 ng/mL concentration, on the other hand, hampers PDLSCs osteogenic development [25]. Our earlier study reveals that trauma-induced inflammation enhanced DPSCs osteogenic differentiation in rats [26]. Remarkably, mild irritations (i.e., 10 ng/mL TNF-α) have been reported to promote osteogenic and odontogenic differentiation of dental pulp cells [24, 27, 28]. The inflammation, defective differentiation, repair, and regeneration processes in the microenvironment and the detailed molecular mechanism remain unknown.

After examining the osteogenic and odontogenic differentiation of iDPSCs and EVs on PDLSCs, high-throughput sequencing and bioinformatics were employed to investigate EV’s miRNA cargoes to uncover the underlying molecular mechanism. We intend to uncover a role for miRNAs in osteogenic and odontogenic differentiation and to investigate the relationship between elevated expression of miRNAs and the conventional osteogenic and odontogenic pathway. This study examined how osteogenic and odontogenic differentiated iDPSCs can influence downstream PDLSCs by delivering specific miRNA-rich EVs.

## Methods

### Study design

**Fig. 1:**
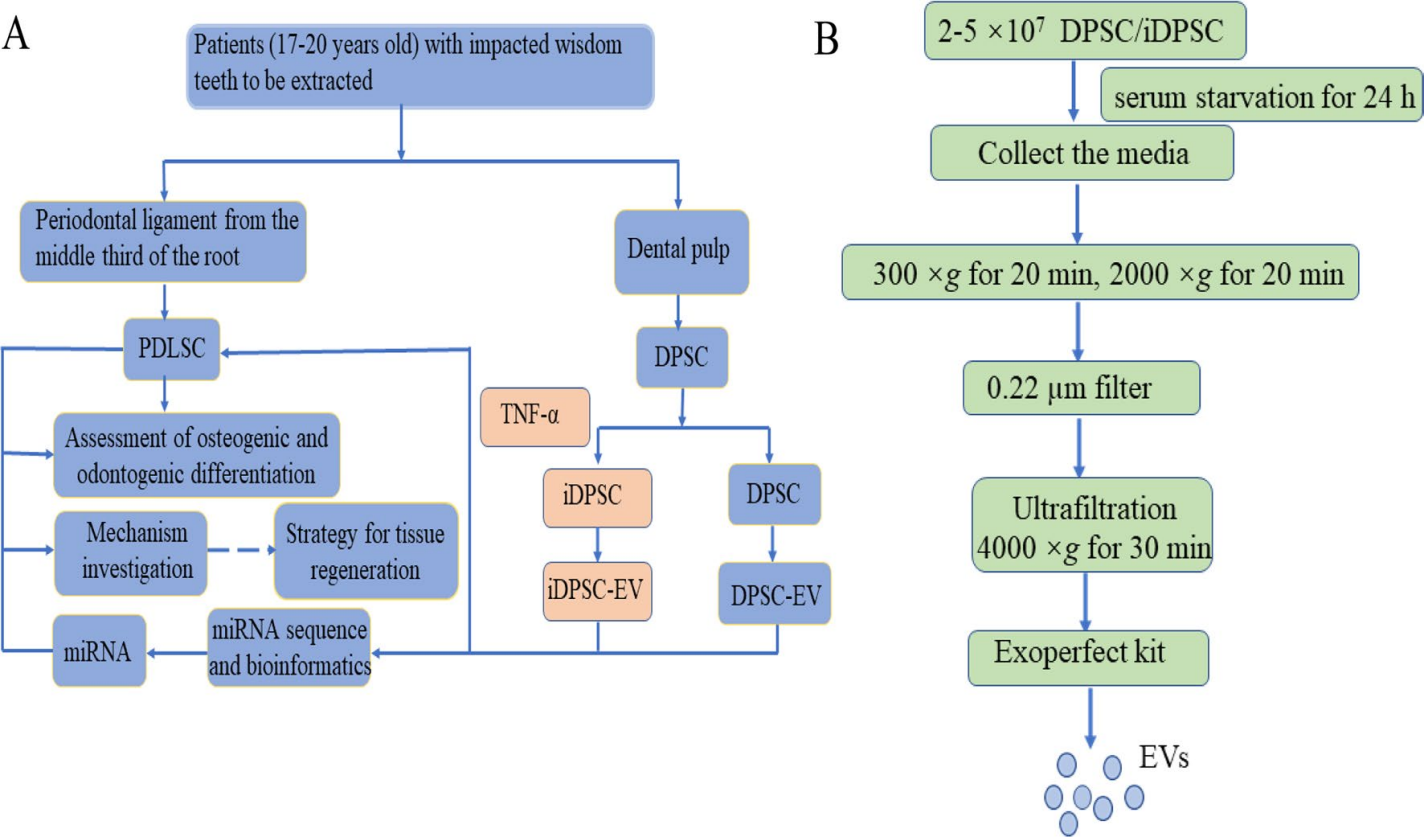
Conceptual schematic about associated flows. **A** Study design. **B** EV’ s isolation

In this basic science research, healthy young patients enrolled in Nanjing Medical University's affiliated stomatology hospital donated their impacted wisdom teeth for isolation of DPSCs and PDLSCs. All patients gave written informed consent, and the ethics committee of Nanjing Medical University approved the study. DPSC were incubated with 10 ng/mL of TNF-α for three days to simulate the known process of dentin repair. The osteogenic and odontogenic differentiation enhancement of iDPSCs and PDLSCs (treated with iDPSC-EV) were tested. After examining miRNAs in EVs using miRNA sequences and experimental confirmation, a presumed major functional miRNA with high expression was chosen for downstream validation after its functional test of promoting osteogenic and odontogenic differentiation (Fig. 1A).

### Cell culture

This study followed the Declaration of Helsinki’s standards and was approved by Nanjing Medical University’s ethical committee. 9 Donors without systemic diseases provided informed consent that they were patients (age, 17–20 years, 5 males and 4 females) at a stomatology facility connected with Nanjing Medical University who needed a wisdom tooth extraction. The periodontal ligament tissue was obtained from the middle third of the root surface, and the picked-out dental pulp was cut into small pieces. Both types of tissues were collected in 500 µL alpha minimum essential medium (α-MEM, Gibco, USA) supplemented with 500 µL 3% type I collagenase (Sigma, USA) and 200 µL 0.25% trypsin (BI, Israel) for 30 min digestion. Following the digestive reaction, the tissue was resuspended in 3 mL of culture medium containing α-MEM, 10% fetal bovine serum (FBS, Gibco, USA), 100 mg/mL of streptomycin, and 100 units/mL of penicillin (Gibco, USA). Cells grew in a humidified atmosphere with 5% CO_2_ at 37 °C, and the culture medium was refreshed every other day. Passage 3–5 were used for experiments. When confluence reached 60%, α-MEM containing 10 ng/mL TNF-α (300-01A-50, PeproTech, USA) was used to induce DPSC for 3 days. The step was designed to gain iDPSC.

### Identification of surface markers by flow cytometry

This experiment was carried out to determine the cell's mesenchymal origin. 1 × 10^4^ cells were digested by 0.25% EDTA-free trypsin and resuspended in PBS. Along with the instruction, they were incubated with CD34, CD45, CD29, CD73, CD90, and CD105 (BD pharmingen, Italy) in the dark on the ice for 1 h. Then the cells were washed with PBS twice and subjected to a FAC Scan flow cytometer (BD Biosciences, USA).

### Alizarin red S (ARS) staining

The experiments in this section were designed to assess calcium-rich deposits or osteogenic differentiation. Cells were cultured in 12-well or 6-well plates and induced for 21 days by mineralization induction medium (MM), which was composed of a culture medium supplemented with 50 μM ascorbic acid, 10 nM dexamethasone, and 10 mM β-glycerophosphate (Sigma, USA). After fixation by 75% alcohol for 30 min, cells were washed with deionized water and stained with ARS (Sigma, USA) for 5 min at room temperature. The Microscope was used for observation and photographing (DMC2900, Leica, Germany). For quantification analysis, OD values of the solution at 560 nm wavelength were recorded after the mineralized nodules were completely eluted by 10% CPC (Sigma, USA) for 30 min. The final result was normalized to protein quantification by BCA kit (P0012S, Beyotime, China).

### Adipogenic differentiation assay

This experiment was designed to determine MSC's adipogenic differential potential, which is one of the MSC’s multi-lineage differentiation. Cells were seeded in 6-well plates and grown in an adipogenic induction medium (Cyagen Biosciences Inc., CA, USA) according to the manufacturer's instructions. One month later, the cells were fixed with 4% paraformaldehyde for 30 min and stained with Oil red O solution at room temperature for 30 min. The microscopy photographs were captured and recorded.

### Chondrogenic differentiation assay

Multi-lineage differentiation characteristics include chondrogenic differentiation. 1 × 10^5^ cells were centrifuged at 1000 rpm for 5 min, and the cell pellets were cultured in a chondrogenic differentiation medium (Cyagen Biosciences Inc. CA, USA) in 15 mL conical-bottomed sterile centrifuge tubes. The medium was changed every other day. After 1 month, the pellets were fixed and embedded in OCT (SAKURA, Japan) compound. Frozen sections (5 μm thickness) were stained with Alcian blue solution and observed by microscope.

### CCK-8 assay

The CCK-8 test kit (Dojindo, Japan) was used to analyze cell proliferation according to the manufacturer's instructions. A total of 2 × 10^3^ cells per well were plated in a 96-well plate, after serum starvation for 24 h,10 µL reagent plus 90 µL α-MEM per well was added and incubated at 37 °C for 2 h according to the instructions. Data were collected after 0, 1, 3, 5, and 7 days of culture, and the test was repeated another 2 times.

### Flow cytometry analysis of cell cycle

This method was employed to analyze the growth kinetics of DPSC. After induction and transfection, the cells were digested with EDTA-free trypsin and washed with PBS twice. The sediment was fixed by 1 mL 70% alcohol under − 20 °C overnight. After being washed with PBS 3 times, cells were dyed with 500 μL propidium iodide (PI, 550825, BD, USA) and incubated for 15 min at room temperature before analysis on a FACScan flow cytometer (BD Biosciences, USA).

### Quantitative reverse transcription and polymerase chain reaction (qRT-PCR)

This experiment was performed to examine the relative expression of RNAs. TRIzol™ (Invitrogen, USA) was used to isolate total RNA. 1 µg of RNA, RT Reagent Kit (Vazyme, China), and other materials were used for reverse transcription (RT). qRT-PCR was performed using QuantStudio™ 7 Flex Real-Time PCR Systems (ABI, USA) and Roche Light Cycler 480 sequence detection system (Roche Diagnostics, Switzerland). The procedure (pre-denaturation, cycles of denaturation, and extension) was set based on underlined parameters: 95 °C for 30 s, 95 °C for 10 s and 60 °C for 30 s (40 cycles), 95 °C for 15 s, 60 °C for 1 min, and 95 °C for 15 s. Primers used in this experiment are listed in Table 1. The threshold cycle (CT) value was used to measure the expression of genes (*ALP*, *DSPP*, *RUNX2*, *LMBR1*, *OSX*, *GAPDH*, *U6*, and miR-758-5p). *GAPDH* normalized the mRNA expression, and *U6* was the internal control of miR-758-5p. The converted fold changes (2^−ΔΔCt^) and results were shown as an n-fold difference relative to the control, as previously described [29].

**Table 1:** Gene-specific reference primer sequences utilized for qRT-PCR analysis

Gene	Sequence
*GAPDH*	F: TCAACAGCGACACCCACTC
	R: GCTGTAGCCAAATTCGTTGTC
*ALP*	F: CCAAAGGCTTCTTCTTGCTG
	R: CCACCAAATGTGAAGACGTG
*RUNX2*	F: TCGCCAGGCTTCATAGCAAA
	R: GGCCTTGGGTAAGGCAGATT
*DSPP*	F: ATATTGAGGGCTGGAATGGGGA
	R: TTTGTGGCTCCAGCATTGTCA
*OSX*	F: CCTCCTCAGCTCACCTTCTC
	R: GTTGGGAGCCCAAATAGAAA
*LMBR1*	F: GCGGGAGTCCACGATATGTTT
	R: GCTGACACTGCGAGAGTGAA
miR-758-5p	F: GCGGATGGTTGACCAGAGA
	R: AGTGCAGGGTCCGAGGTATT
*U6*	F: CTCGCTTCGGCAGCACA
	R: AACGCTTCACGAATTTGCGT

### Western blot

The assay represented the relative protein expression for osteogenic and odontogenic differentiation markers, miRNA’s target, and pathway protein. The total protein was isolated by RIPA lysis buffer (Beyotime, China) containing 1 mM PMSF (Beyotime, China). The protein sample was loaded onto 10% SDS-PAGE gel for electrophoresis, and then transferred onto 0.45 mm PVDF membranes (Millipore, USA) at 300 mA in a blotting apparatus (Tanon, China). Membranes were blocked with 2% non-fat milk for 2 h, then incubated in primary antibody overnight. The information of primary antibodies are as follows, RUNX2 (#12556, cell signaling technology, USA and ab23981, Abcam, UK), ALP (ab65834, Abcam, UK), OSX (ab22552, Abcam, UK), BMP2 (AF5163, Affinity, USA), BMP4 (BF0696, Affinity, USA and 12492-1-AP, proteintech, USA), LMBR1 (A18484, Abclonal, China), DSPP (BS71212, Bioworld, USA), GAPDH (10494-1-AP, proteintech, USA); Professor Jiang Hongbing of Jiangsu Province Key Laboratory of Oral Disease generously donated antibodies of CD63, CD9, and CALNEXIN. Next, the membranes were incubated in HRP-conjugated secondary antibody for 1 h, and signals were detected by enhanced chemiluminescence reagent (WBULS0100, Millipore, USA). Image J was used for the grayscale analysis of western blot.

### Isolation of EVs

DPSC were serum-starved for 24 h prior to isolation of the EVs. EVs were isolated along with the instruction of the Exoperfect kit (EXOMU10A-1, Sesh-biotech, China) and purification by ultrafiltration test tube (UFC910096, Millipore, USA). To accomplish condensation, the collected culture media were centrifuged at 300×*g* for 10 min and 2000×*g* for 20 min, and then passed through a 0.22 µm filter (SLGP033RB, Millipore, USA) before being transferred to an ultrafiltration tube and centrifuged at 4000×*g* for 30 min [30]. 1 mL condensation was combined with 200 µL reagents and incubated overnight at 4 °C. After 30 min of centrifugation at 1500×*g*, the supernatant was discarded and the remaining pellet was centrifuged at the same speed for another 5 min to obtain the final product (Fig. 1B). Then, 500 µL PBS was used to resuspend the precipitation, and protein quantification was performed using the bicinchoninic acid (BCA) assay. The finished EVs solution was kept at 4 °C for 1 week or at − 80 °C for an extended time [31].

### Characterization of EVs

According to the guideline of JSEV [32], morphological characteristics were shown through TEM pictures (Hitachi TEM system, Japan), the diameter was evaluated by NTA (snqbio, China) and protein marker (CD63 and CD9 represented the positive marker, and CALNEXIN which expresses in endoplasmic reticulum was the negative marker for exosome) were tested by western blot in EVs and parent cells.

### EVs phagocytosis

This experiment was used to demonstrate the PDLSCs endocytosis of EVs. According to the instruction, 1 µL pkh26 (BB-441125, BestBio, China) in 250 µL diluted C (PBS) incubated EVs for 5 min at room temperature. Then 1% BSA in the same volume as the mixture was used to end the reaction. The resuspended EVs were added to the culture medium of PDLSC. After 24 h, the cell slides were collected, washed with PBS, and fixed with 4% paraformaldehyde. After being dyed with 2-(4-Amidinophenyl)-6-indolecarbamidine dihydrochloride (DAPI) (C1006, Beyotime, China), the slides were visualized on the stage of the fluorescence microscope (DM4000, Leica, Germany).

### ALP activity assay

This experiment was used to select optimal EVs’ concentration for mineralization. After induction for 7 days, the cells were lysed in 0.5% Triton X-100 (Beyotime, China) for 30 min. ALP quantitative analysis was performed following the protocol using an alkaline phosphatase assay kit (Jian Cheng, China). 30 µL lysis solution mixed with 50 µL reagent I and 50 µL reagent II, then incubated in 37 °C water bath for 30 min following mix with 150 µL reagent III. OD values were read at 520 nm. This experiment was performed in triplicate.

### EVs treatment

PDLSCs were treated with different EVs at optimal concentration for 3 days and 7 days to examine EV's differential effect. The groups were marked as D0, D3 with DPSC-EV, D3 with iDPSC-EV, D7 with DPSC-EV, and D7 with iDPSC-EV. The evaluation was done by time and intergroup contrast at the same time point [33, 34]. In this part and EVs phagocytosis, we used EVs-free FBS (EXOFBSS0A-1, ExoPerfect, China) to prepare the culture medium, and PBS in the same volume as EVs solution was added to the culture medium of the D0 group.

### High-throughput miRNA sequencing and enrichment analysis of transcription binding motifs

This part was conducted by CapitalBio Technology and analyzed the miRNA’s content. Total RNA was isolated from the EVs using the TRIzol™ reagent (Invitrogen, USA), and a library was constructed on the Illumina Platform. Before formal analysis, the raw data underwent quality control and data filtering. The evaluation of differential expression was processed with *P*-value and fold-change (FC) as a criterion (*P* < 0.05 and log_2_FC > 0.5). The target genes were predicted by miRDB and miRwalk software, and the following enrichment analysis was based on GO (Gene Ontology) and KEGG (Kyoto Encyclopedia of Genes and Genomes) databases. The disease analysis was based on the KEGG Disease databases, which included all of the human diseases that had been recorded, and was also intended to verify the activities of target genes. The R language was used for data analysis and output.

### Transfection assay and gene-targeting

To investigate the impact and similar EV-like behavior of miR-758-5p on PDLSCs. The PDLSCs were transduced with miR-758-5p mimic, inhibitor, and negative controls (mimic NC and inhibitor NC). To demonstrate the role of *LMBR1* in osteogenic and odontogenic differentiation of PDLSCs, *LMBR1* was stably silenced by small interfering RNA (siRNA) compared to its corresponding negative control (si NC). After 72 h, the transfection reagents-containing medium was changed to a normal culture medium. The underlined reagents were provided by Ribobio (Guangzhou, China).

### Immunofluorescence staining

This experiment was used to verify the results of the western blot. The PDLSCs were grown on coverslips for 24 h before being fixed with 4% paraformaldehyde and blocked for 1.5 h at 37 °C with goat serum. After incubation with primary antibody at 4 °C overnight and washed by PBS 3 times, the cells were incubated with cy3 conjugated secondary antibody at room temperature for 1 h. After DAPI-staining for 5 min, the expression level was recorded using a fluorescence microscope.

### Dual-luciferase reporter assay

This assay was used to identify the directed combination of miRNA and target. The wide-type and mutant binding sites for the miR-758-5p genomic region of LMBR1 plasmids (LMBR1-WT and LMBR1-MUT) were constructed and harbored by a luciferase reporter gene vector (Genechem, China). One of the LMBR1 plasmids and miR-758-5p mimic or mimic NC were co-transfected into 293T cells via Lipofectamine 2000 (Invitrogen, USA) for 6 h in a 24-well plate. Luciferase activity of Renilla and Firefly was detected using a dual-luciferase reporter assay system (Promega, USA) at 48 h after transfection. The data were represented as a ratio, and Renilla served as an internal control.

### Statistical analysis

All quantitative data were presented as the mean and standard deviation (mean ± SD) and analyzed by Student’s t-test or one-way analysis of variance (ANOVA). The statistical analysis was carried out using SPSS 20.0 software. *P* < 0.05 was considered statistically significant.

## Results

### Cell culture and identification

**Fig. 2:**
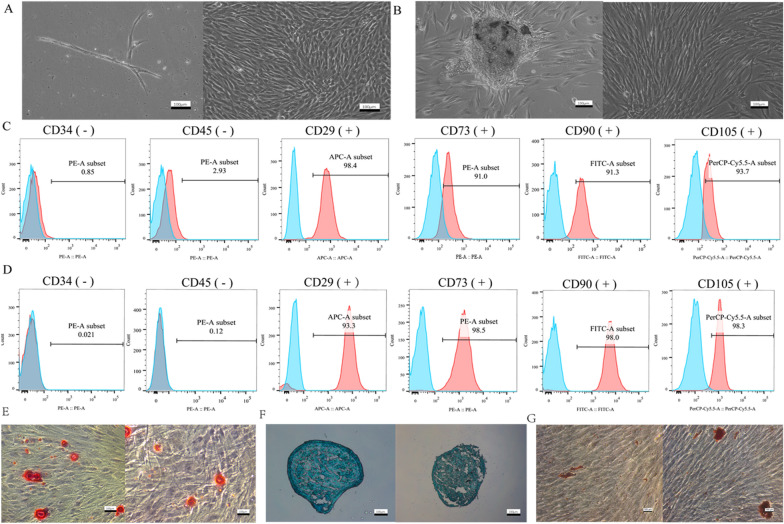
Cell culture and identification. **A** Primary and passage 4 DPSCs from left to right. **B** Primary culture and 4th passage PDLSCs from left to right. **C** Surface markers of DPSCs were analyzed by flow cytometry. **D** Surface markers of PDLSCs were tested by flow cytometry. The blue part represented the control (unstained cell). **E** Result of ARS staining of DPSCs and PDLSCs from left to right. **F** Result of Alcian blue staining of DPSCs and PDLSCs from left to right. **G** Result of Oil red O staining of DPSCs and PDLSCs from left to right. Scale bar, 100 μm

The DPSCs and PDLSCs have a spindle-like form and are placed in a whirlpool pattern (Fig. 2A, B). CD29, CD73, CD90, and CD105 were positive for mesenchymal stem cell markers, whereas CD34 and CD45 were negative (Fig. 2C, D). DPSCs and PDLSCs have osteogenic, chondrogenic, and adipogenic differentiation potential, as shown by Alizarin Red S (Fig. 2E), Alcian blue staining (Fig. 2F), and Oil Red O staining (Fig. 2G).

### Isolation and identification of iDPSC-EV

**Fig. 3:**
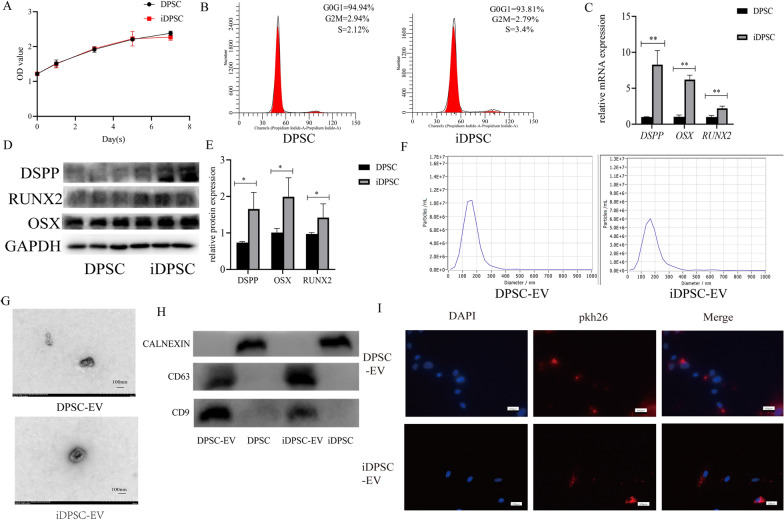
Isolation and identification of iDPSC-EV. **A** Results of CCK-8 assay. **B** Results of Flow cytometry analysis of cell cycle. **C** Relative mRNA expression of *DSPP*, *OSX,* and *RUNX2* was demonstrated by qRT-PCR. **D** The protein levels of DSPP, OSX, and RUNX2 was determined by western blot. **E** The quantification of band intensities was shown by a histogram with the manner in the ratio of the target protein to GAPDH. **F** NTA showed the size distributions of these EV. **G** TEM photos of EVs, scale bar, 100 μm. **H** The expression of CD9, CD63 and CALNEXIN in EV and parent cells. **I** The result of EV phagocytosis, scale bar, 100 μm. n = 3, **P < 0.01, *P < 0.05

The CCK-8 assay and cell cycle analysis revealed that the inflammatory milieu did not significantly increase proliferation (Fig. 3A, B). TNF-α induction at 10 ng/mL for 3 days led to significant changes in DPSC levels of *DSPP*, *OSX*, and *RUNX2*, as well as associated proteins (Fig. 3C–E). Figure 3G revealed EVs with a caved plate or cup-like appearance, with a diameter distribution mainly below 200 nm and a peak of 163 nm for DPSC-EV and 171.2 nm for iDPSC-EV (Fig. 3F). With the final volume of 500 µL (corresponding to secreted by 2–5 × 10^7^ cells), the concentration (by NTA and protein quantification) was 1.3 × 10^10^ particles/mL and 1.63 mg/mL for DPSC-EV and 2.7 × 10^10^ particles/mL and 2.01 mg/mL for iDPSC-EV. The western blot assay indicated that EVs express CD9 and CD63 but not CALNEXIN. Although the band intensity of CD9 and CD63 in parent cells was low, CALNEXIN expression was high (Fig. 3H). PDLSCs plasma shed the red fluorescence of pkh-26 after co-culture with pkh26-labeled EVs, indicating that EVs were successfully absorbed by PDLSCs (Fig. 3I).

### EVs affected the osteogenic and odontogenic differentiation of PDLSCs

**Fig. 4:**
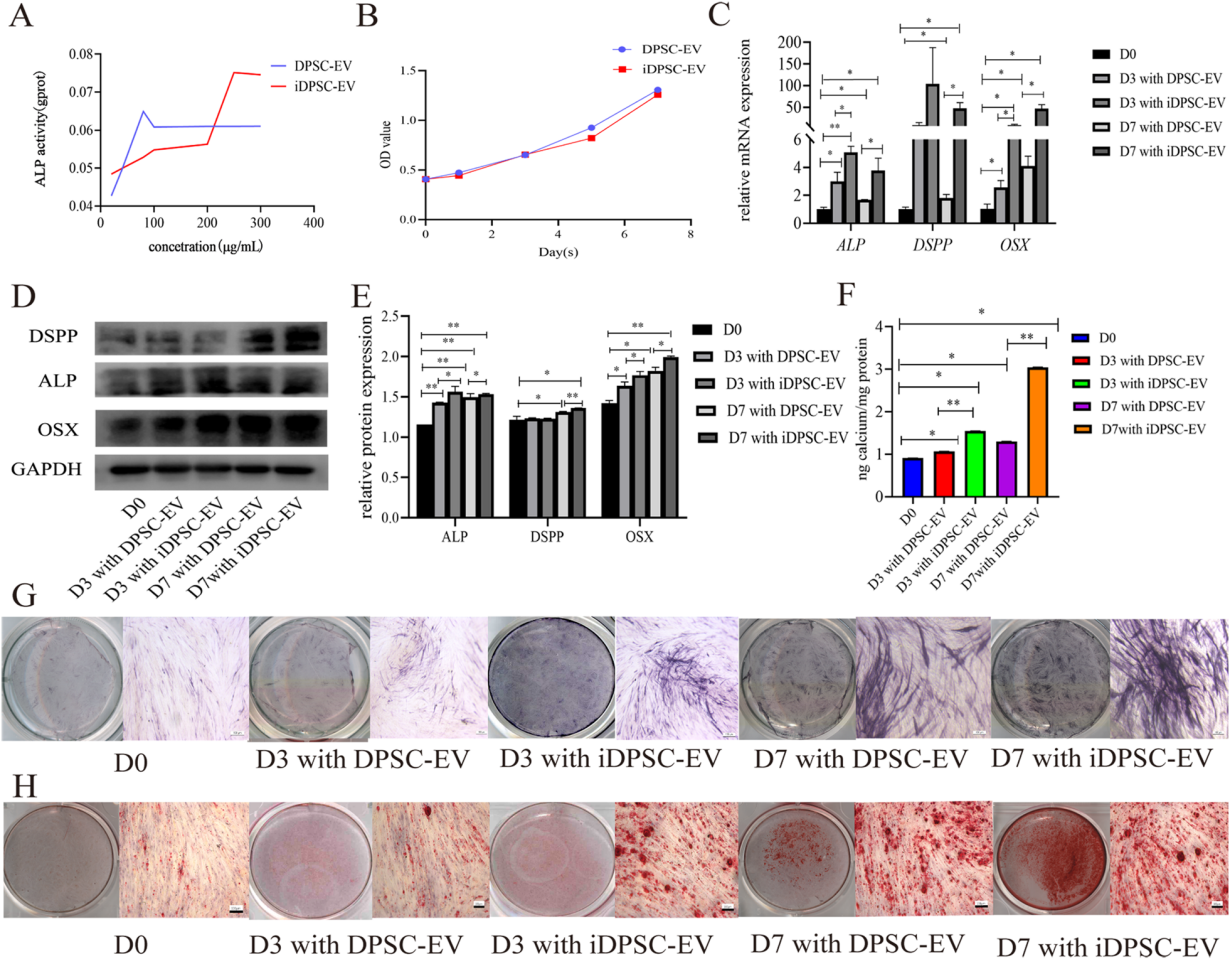
EVs promoted PDLSCs osteogenic and odontogenic differentiation with no significant intergroup proliferative difference. **A** ALP activity of PDLSCs induced by different concentrations of EV for 7 days. **B** CCK-8 revealed the impact on cell proliferation from DPSC-EV and iDPSC-EV. **C** The relative mRNA levels of *ALP*, *OSX,* and *DSPP* after induced by EV at different time points. **D** The western blot bands showed EV-mediated expression of ALP, OSX, and DSPP. **E** The histogram showed the relative quantitative analysis of western blot. **F** Result of quantification of ARS. **G** Result of ALP staining. **H** Result of ARS staining. n = 3, **P < 0.01, *P < 0.05. Scale bar, 100 μm

When varied gradient concentrations of EVs induced PDLSCs, the ALP activity test revealed a concentration-dependent mode that was saturable with the top at 80 µg/mL for DPSC-EV and 250 µg/mL for iDPSC-EV (Fig. 4A). PDLSCs demonstrated increased ALP activity as a result of the increased cellular absorption concentration, and the top of the iDPSC-EV group was found higher than the DPSC-EV group. When the CCK-8 assay was used, there was no discernible change in cell viability between the groups (Fig. 4B). According to data obtained from qRT-PCR (Fig. 4C) and western blot (Fig. 4D, E), PDLSCs by iDPSC-EV inducement strongly expressed OSX and ALP compared to the group induced by DPSC-EV at the same time point. Enhancing odontogenic differentiation required further time. During the first 3 days, no significant change in expression of DSPP was observed; Significant upregulation was seen at D7 with the intergroup difference. The ARS staining (Fig. 4H) and quantification (Fig. 4F) showed that calcium nodules accumulate over time, with more calcium nodules appearing in the groups exposed to iDPSC-EV. ALP staining intensity (Fig. 4G) rose similarly to the above results.

### miRNA sequence, target prediction, and bioinformatics analysis of EVs

**Fig. 5:**
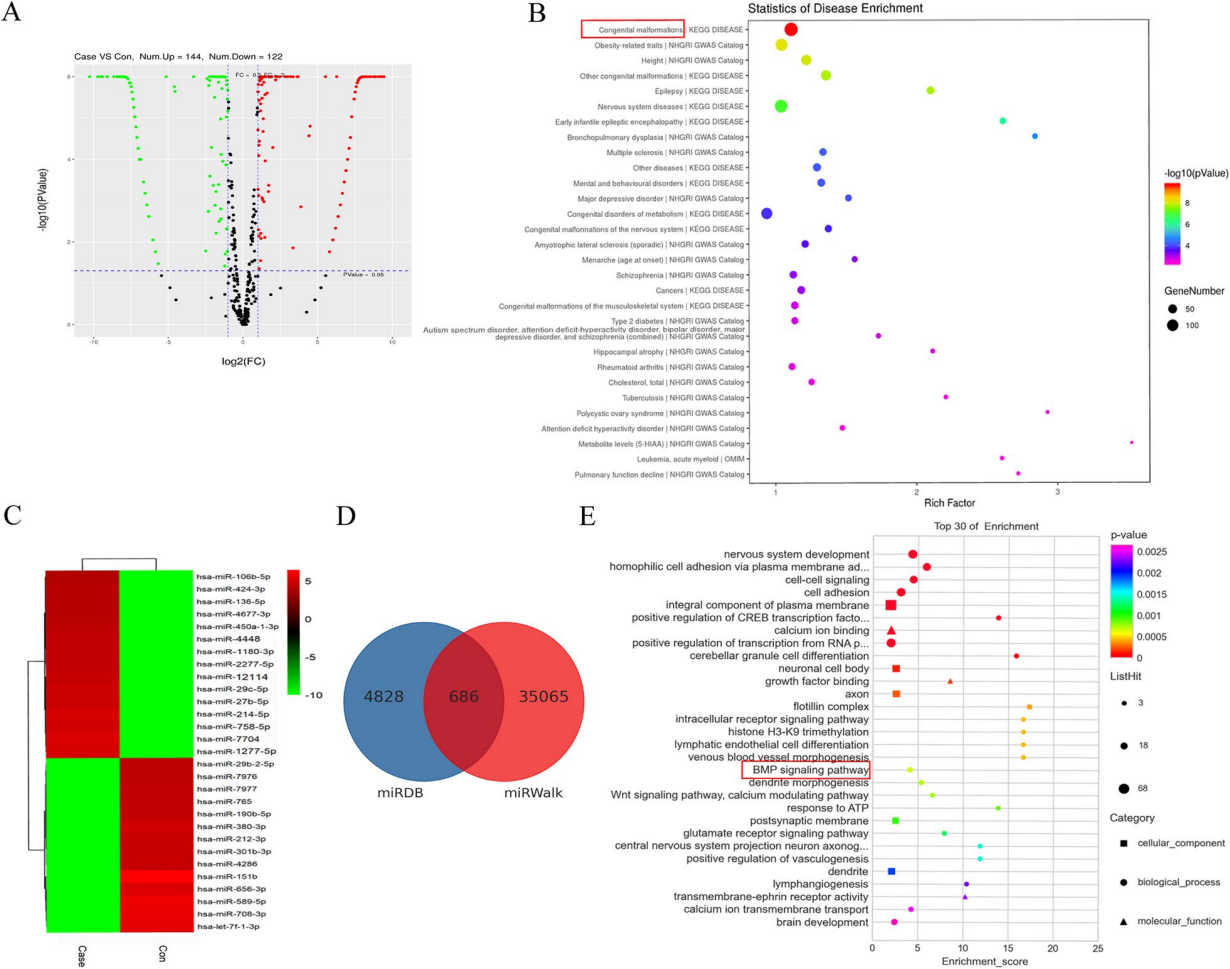
Bioinformatic analysis of differentially expressed miRNA cargoes between iDPSC-EV and DPSC-EV. **A** The volcano figure showed the differentially expressed miRNAs. Case and con represented iDPSC-EV and DPSC-EV. **B** Disease analysis of all the upregulated miRNAs’ targets. **C** Heat map of differentially expressed top 15 up-and down-regulated miRNAs. **D** Target genes of the top 30 miRNAs were predicted by miRDB and miRWalk. **E** Functional analysis of the top 30 miRNAs’ targets by GO enrichment

Between iDPSC-EV and DPSC-EV, there were 144 upregulated miRNAs and 122 downregulated miRNAs, indicating that the miRNA profile in iDPSC-EV was considerably different (Fig. 5A, C). Table 2 showed the top 10 miRNAs that were elevated. The expected targets of known upregulated miRNAs contributed to several functions, for example, the top three include congenital anomalies, obesity-related features, and height, according to Disease analysis (Fig. 5B). The GO enrichment analysis predicted that the top 30 miRNAs’ target genes (Fig. 5D) are involved in a variety of pathways, including differentiation-associated BMP signaling (Fig. 5E). Additionally, Additionally, Wnt signaling pathway was also identified with less significance in the top 30 of KEGG enrichment (Additional file 1). By combining the available literature, we identified miR-758-5p as a highly intriguing candidate for future confirmation. LMBR1 was predicted to be a miR-758-5p target due to its role in congenital malformations.

**Table 2:** The differences in miRNA expression (top 10) between DPSC-EV and iDPSC-EV

miRNA	Log_2_FC	*P* value	Regulation
miR-1277-5p	9.440050376	4.20784E−20	Up
miR-7704	9.321583897	9.40652E−19	Up
miR-758-5p	9.192512248	2.32721E−17	Up
miR-214-5p	9.099567218	1.52753E−16	Up
miR-27b-5p	8.893520877	1.20778E−14	Up
miR-29c-5p	8.837058688	3.27725E−14	Up
miR-12114	8.747992698	1.49345E−13	Up
miR-2277-5p	8.551446826	5.64977E−12	Up
miR-1180-3p	8.515919047	9.60067E−12	Up
miR-4448	8.515919047	9.60067E−12	Up

### EVs affect miR-758-5p, LMBR1, and BMP signaling

**Fig. 6:**
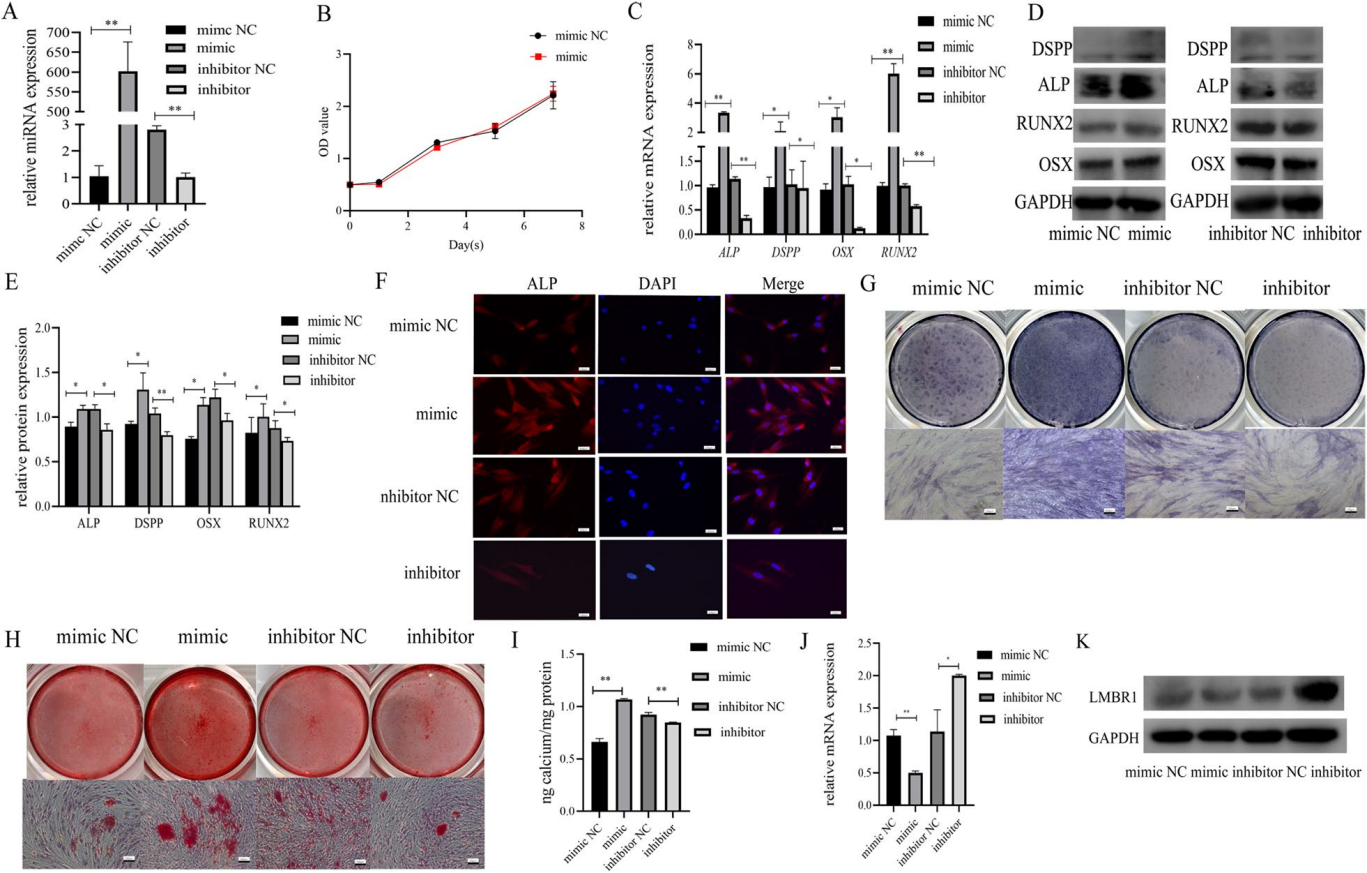
Upregulated miR-758-5p promoted osteogenic and odontogenic differentiation. **A** Transfection efficacy was shown by qRT-PCR in the manner of relative miRNA expression in PDLSCs. **B** CCK-8 assay showed the impact of overexpression of miR-758-5p on proliferation. **C** The relative expression of *ALP*, *DSPP*, *OSX*, and *RUNX2* in the mimic, mimic NC, inhibitor, and inhibitor NC group. **D** Western blot assay detected the expression of ALP, DSPP, RUNX2, and OSX when miR-758-5p was overexpressed or inhibited. **E** Statistic analysis of the grayscale of western blot and data were shown as the ratio of the target protein to GAPDH. **F** Immunofluorescence photos showed that the intensity (represented the expression of ALP) in mimic, mimic NC, inhibitor, and inhibitor NC groups. **G** Result of ALP staining. **H** Result of ARS staining. **I** Quantification of ARS staining. **J** qRT-PCR showed that miR-758-5p regulates *LMBR1*. **K** Western blot showed that miR-758-5p regulates LMBR1. n = 3, **P < 0.01, *P < 0.05. Scale bar, 100 μm

The qRT-PCR was performed to determine the efficacy of the transfection, and the up-and down-regulated levels were satisfactory at 72 h after transfection (Fig. 6A). No significant variation in proliferative potential was observed in PDLSCs overexpressing miR-758-5p (Fig. 6B). To confirm the improved bone and dentin production potential, PDLSCs were transfected with 50 nM mimic NC, 50 nM mimic, 100 nM inhibitor NC, and 100 nM inhibitor. Results from qRT-PCR and western blot displayed that highly-expressed miR-758-5p can modulate osteogenic and odontogenic mRNA (*DSPP*, *ALP*, *OSX*, and *RUNX2*) and related protein levels, and the opposite effect existed in the inhibitor group (Fig. 6C–E). The osteogenic tendency was also found in the immunofluorescence staining study of ALP, with the mimic group emitting more than the mimic NC and the inhibitor group exhibiting decreased fluorescence intensity (Fig. 6F). ALP activity was suppressed by miR-758-5p inhibitor from ALP staining (Fig. 6G). The ARS staining and quantification (Fig. 6H, I) revealed that the mimic group formed more calcified nodules than the mimic NC, whereas the inhibitor showed the opposing results.

**Fig. 7:**
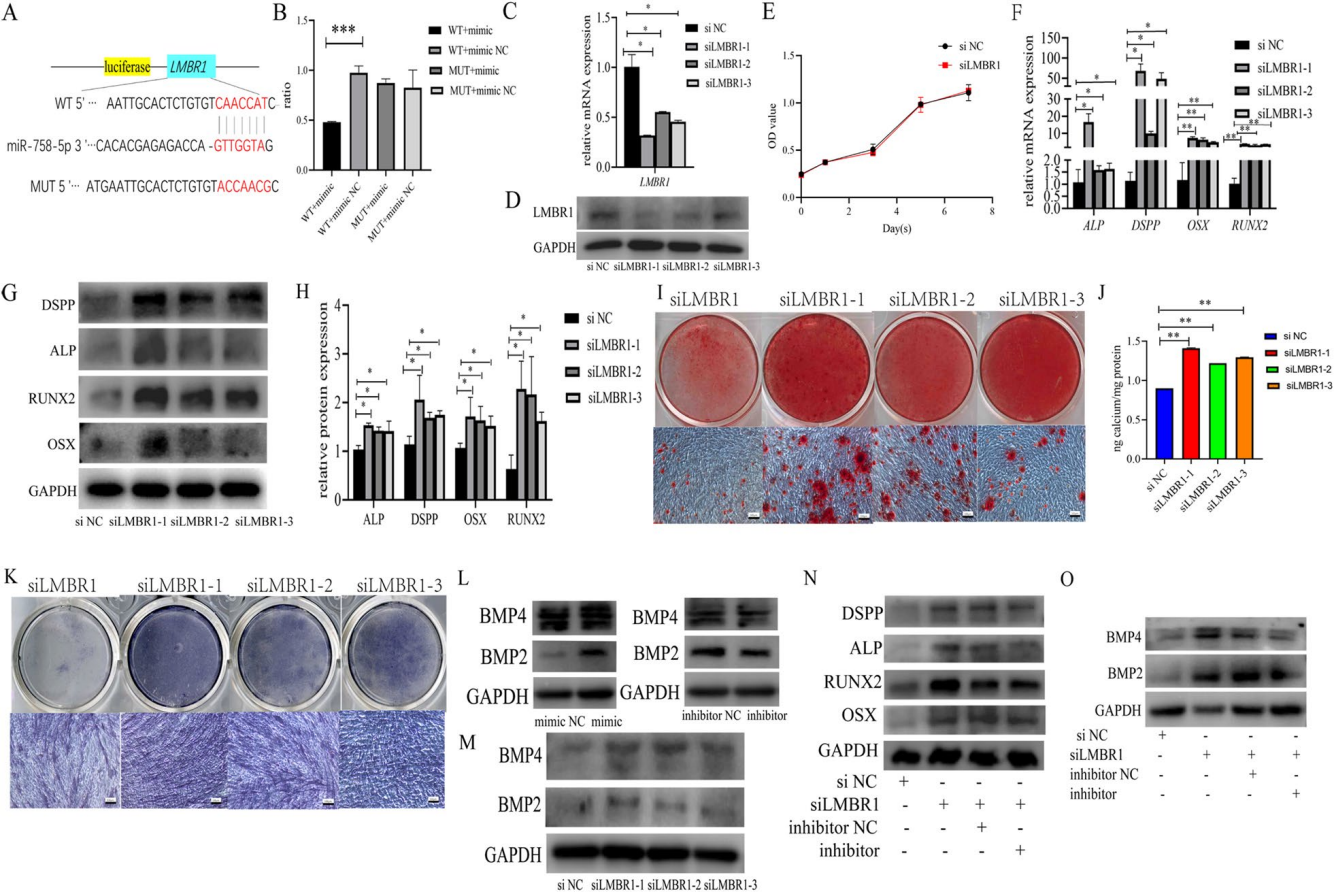
Knock-down of *LMBR1* promoted the osteogenic and odontogenic differentiation of PDLSCs. **A** The potential binding site between miR-758-5p and *LMBR1* predicted by bioinformatics. **B** Dual-luciferase reporter assay demonstrated the correlation between miR-758-5p and *LMBR1*. **C** qRT-PCR evaluated the downregulation of *LMBR1* by siRNAs. **D** Western blot evaluated the suppression of LMBR1 by siRNAs. **E** The results of the CCK-8 assay showed cell proliferation after the downregulation of LMBR1. **F** Relative mRNA levels of *ALP*, *DSPP*, *OSX*, and *RUNX2* when LMBR1 was knocked down. **G** Western blot assay showed the expression of ALP, DSPP, OSX, and RUNX2 after transfection of siRNAs. **H** Histogram derived from relative protein expression of western blot. **I** Result of ARS staining. **J** Quantification of ARS staining. **K** Result of ALP staining. **L** Western blot showed the condition of BMP2 and BMP4 when miR-758-5p was overexpressed or inhibited. **M** Western blot showed the condition of BMP2 and BMP4 when LMBR1 was knocked down. **N** Protein levels of ALP, DSPP, RUNX2 and OSX in rescue experiment. **O** Protein levels of BMP2 and BMP4 in rescue experiment. n = 3, ***P < 0.001, **P < 0.01, *P < 0.05. Scale bar, 100 μm

The qRT-PCR and western blot analysis revealed that miR-758-5p mimics reduced *LMBR1* expression and translation (Fig. 6J, K). Dual-luciferase reporter assay revealed that 293T cells transfected with miR-758-5p mimic and LMBR1-WT produced the least amount of luciferase signal (Fig. 7A, B).

Three LMBR1 siRNAs (siLMBR1, designated as siLMBR1-1, siLMBR1-2, and siLMBR1-3) and negative control (si NC) were utilized to examine the relationship between LMBR1 and osteogenic and odontogenic differentiation. siLMBR1-1 was chosen for the following experiments due to its excellent knockdown efficiency (Fig. 7C, D). *LMBR1* silencing did not affect the growth of PDLSCs (Fig. 7E). LMBR1 deficiency increased osteogenic and odontogenic mRNA and protein expression (Fig. 7F–H), contributed to the development of mineralized nodules as determined by ARS staining and quantification (Fig. 7I, J), and increased the intensity of ALP staining (Fig. 7K), indicating increased ALP activity.

**Fig. 8:**
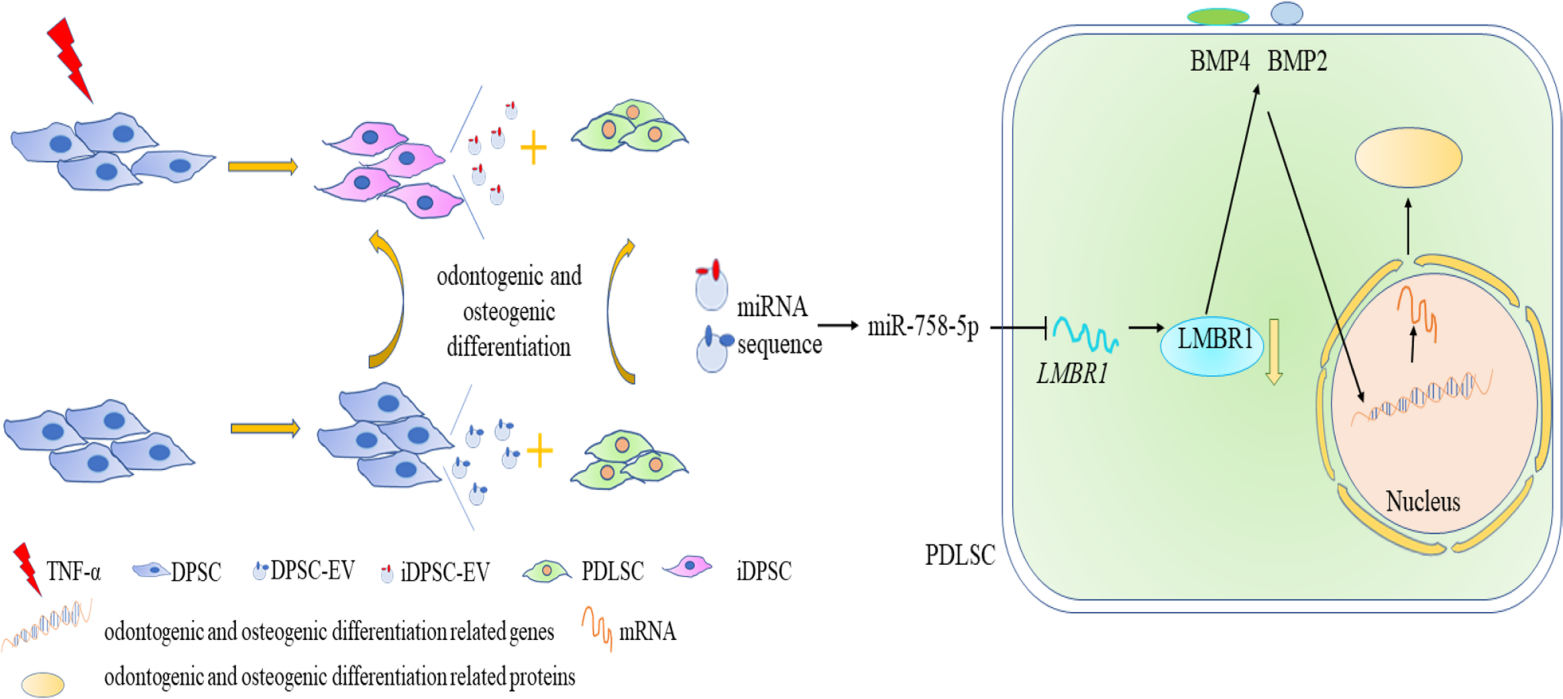
Schematic diagram. The miR-758-5p/LMBR1/BMP2/4 axis participates in EV-mediated osteogenic and odontogenic differentiation

Mechanically, miR-758-5p derived from EVs facilitated signal transduction in PDLSC and stimulated BMP signaling, as shown by the synthesis of BMP2 and BMP4 proteins (Fig. 7L). LMBR1 silencing also affected BMP signaling (Fig. 7M). In rescue experiments, inhibition of miR-758-5p reverted osteogenic and odontogenic differentiation induced by *LMBR1* silencing of PDLSCs (Fig. 7N) and stimulation of BMP signaling (Fig. 7O). Our findings show that iDPSC-EV transports the LMBR1-targeting miR-758-5p to promote PDLSCs osteogenic and odontogenic differentiation via BMP signaling (Fig. 8).

## Discussion

Hard tissue formation, like osteogenesis, needs multi transcription factors and signaling molecules [35]. Communication between cells and their microenvironment activates selective packing and miRNA-mRNA regulatory mechanisms. For example, miRNA transport was facilitated by RNA binding proteins and endosomal sorting complexes required for transport (ESCRT) [36, 37]. Enrichment of pathway analysis revealed an upregulation of signaling, which may provide a compelling explanation. The size of exosomes has not been strictly separated, resulting in different ranges: 30–200 nm, 30–100 nm, and 30–150 nm, and most researchers favor the smallest range [6, 38, 39]. Although we noticed the characteristic caved plate and cup-like shapes and positive expression of protein markers (CD63 and CD9), we referred to the isolation as EVs for the NTA result as having a size distribution profile with peak diameters of 163 and 171.2 nm [32]. The larger size is mostly caused by harvest-related co-precipitation with impurity, which mainly comprises proteins [10, 31]. As described here, the exosomes and EVs we isolated have similar functions if compacting complex plasma payloads into bilayer membrane and in a similar developing manner and digestion process [40]. Additionally, the finding that iDPSC-EV produced more than DPSC-EV validates previous research indicating that inflammatory cytokines mediate paracrine signaling [23].

EVs can be transferred through biofluid or directly target the impact cell in the same microenvironment. A fluorescent tracer was used to confirm that PDLSCs absorbed EVs. According to the ALP activity assay, the biological mineralization increased in a dose-dependent manner [7]. The ALP activity did not alter considerably when the dosage of EVs was larger than the threshold level. PDLSCs ate more iDPSC-EV than DPSC-EV, indicating increased mineralization potential, consistent with the prior finding that EVs from MSCs favor PDLSCs for regeneration [41, 42]. We chose to observe the third and seventh days of inducement to illustrate a dynamic detin and bone formation process [33, 34].

Interestingly, tooth-specific DSPP rose somewhat but not significantly on the 3rd day, despite prior work demonstrating that translation of DSP in DPSCs began 3 days after endocytosis of exosomes from DPSCs [43]. This discrepancy can be explained by EVs separation procedures and different target cells because PDLSC is a possible candidate for periodontal tissue regeneration, reports of application for dental pulp regeneration are quite a few [14]. The entire process included the transcription and translation of *OSX* and *ALP*, which further elucidated PDLSC’s favorable osteogenic characteristics. On the 7th day, there was a clear upregulation of DSPP, which might be explained in part by the promotion caused by increasing OSX expression during development [44]. However, mutation of *DSPP* also changes the expression of osteogenic mRNAs in the alveolar bone cells [45].

The molecular mechanism behind PDLSC osteogenic and odontogenic differentiation mediated by EV is unknown. EVs always convey miRNA to control the differentiation of downstream cells into distinct lineages [43]. Among the miRNAs that own the potential for bone-formation regulation, miR-758-5p sharing the same precursor molecule with the osteogenic miR-758 drew our attention [46]. By combining the sequencing data, predicted target genes, and a literature study, it has been revealed that two of the top ten upregulated miRNA, miR-27b and miR-1277 have an association with inflammation [47, 48]. Functions of other top 10 miRNAs can be explained by complex content of EV and osteoclast-osteoblast communication during bone formation and bone homeostasis maintenance [49].

The miR-758-5p/LMBR1/BMP2/4 axis was predicted using miRNA sequencing, and differential analysis of predicted miRNA and target genes and associated pathways since the members’ functions were associated with the top iDPSC-EV pathways. The effect of miR-758-5p overexpression and knockdown on osteogenic and odontogenic traits in PDLSC was investigated. The dual-luciferase reporter assay further establishes that miR-758-5p directly regulates *LMBR1*. Similarly, silencing LMBR1 enhanced the production of osteogenic and odontogenic genes and proteins.

LMBR1 is a transmembrane protein that associates with the lysosomal membrane and is a potential gene for stemness and skeletal system development [50–52]. In contrast, congenital abnormalities (including skeletal) are at the top of the Disease analysis of iDPSC-EV. Additionally, it has been suggested to promote BMP signaling [51]. Osteoblasts are differentiated from stem cells via a complex gene-signaling system that includes members of the BMP family [53]. Simultaneously, western blot analysis was used to assess the activation of BMP2 and BMP4 which is related to the BMP signaling following transfection. 10 ng/mL TNF-α induced osteogenic and odontogenic differentiation in DPSC and stimulated the secretion of miR-758-5p-encapsulated EV by DPSC, activating downstream signaling pathways involving BMP signaling in PDLSC. BMP2 is always used as an inducer of biological mineralization and is required for repair, and the DPSC-EV can raise it as previously reported [5, 54, 55]. 16 members in the BMP family are mainly involved in bone repair, growth, and skeletal disorders [53]. Increased BMP2 protein expression confirms the activation of BMP signaling [56]. BMP4 has been detected in the odontogenesis of PDLSC and is committed to skeletal development [14, 53]. Similarly, LMBR1 also participates in the development and is negatively related to odontogenesis and osteogenesis by our investigation [57]. BMP2/4 signaling, as previously stated, controls tooth development [58]. Using a systematic approach, we identified *LMBR1* as a downstream target of EV-origin miR-758-5p in the regulation of osteogenic and odontogenic development. Notably, siLMBR1’s mineralization effect may be partially reversed by suppressing miR-758-5p.

It is still a preliminary stage of comprehensive comprehension, and this research has several limitations. Because EV always compact their cargoes from the cell plasma, in-situ hybridization assays, which are a method for miRNA functional study, may be omitted from studies devoted to EV-derived miRNA research [29]. EV are recognized as a non-immune delivery vehicle that specifically loaded miRNA to enhance odontogenesis and osteogenesis [43, 56]. Nonetheless, more work is still needed before stem cells and their derivatives can be fully utilized. The way forward has been paved through surface alteration and scaffolding [59, 60].

## Conclusions

These findings established a unique EV-to-cell communication axis that runs directly between the inflammatory microenvironment and the differentiation of PDLSC. The obtained data suggested that miR-758-5p derived from EV may represent a novel starting point for healing and regenerating oral-facial tissue defects. This is the first time a link has been found between LMBR1 and osteogenic and odontogenic differentiation. As a result, we believe that miR-758-5p-containing EV’ involvement in promoting osteogenic and odontogenic differentiation in PDLSC also serves as a ‘fertile soil’ for ‘seed’.

## Supplementary Information

**Additional file 1.** KEGG enrichment analysis of top 30 miRNAs.

## Data Availability

Data used and analyzed during the current study are available from the corresponding author on reasonable request.

## Abbreviations

EV: Extracellular vesicle
iDPSC-EV: Extracellular vesicle from inflammatory dental pulp stem cell
PDLSC: Periodontal ligament stem cell
qRT-PCR: Quantitative reverse transcription and polymerase chain reaction
ALP: Alkaline phosphatase
ARS: Alizarin red S
CCK-8: Cell Counting Kit-8
TEM: Transmission electron microscopy
NTA: Nanoparticle tracking analysis
MSC: Mesenchymal stem cell
OSX: Osterix
DSPP: Dentin sialophosphoprotein
RUNX2: Runt-related transcription factor 2
CPC: Cetylpyridinium chloride
BCA: Bicinchoninic acid
FC: Fold-change
LMBR1: Limb development membrane protein 1
α-MEM: Alpha minimum essential medium
FBS: Fetal bovine serum
DAPI: 2-(4-Amidinophenyl)-6-indolecarbamidine dihydrochloride
CT: Threshold cycle
GO: Gene Ontology
KEGG: Kyoto Encyclopedia of Genes and Genomes
ESCRT: Endosomal sorting complexes required for transport
