# Supplementary figures and images for: Extracellular vesicles from the inflammatory microenvironment regulate the osteogenic and odontogenic differentiation of periodontal ligament stem cells by miR-758-5p/LMBR1/BMP2/4 axis
Source: J Transl Med. 2022 May 13;20:208. doi: 10.1186/s12967-022-03412-9 (PMC9103284; doi:10.1186/s12967-022-03412-9)

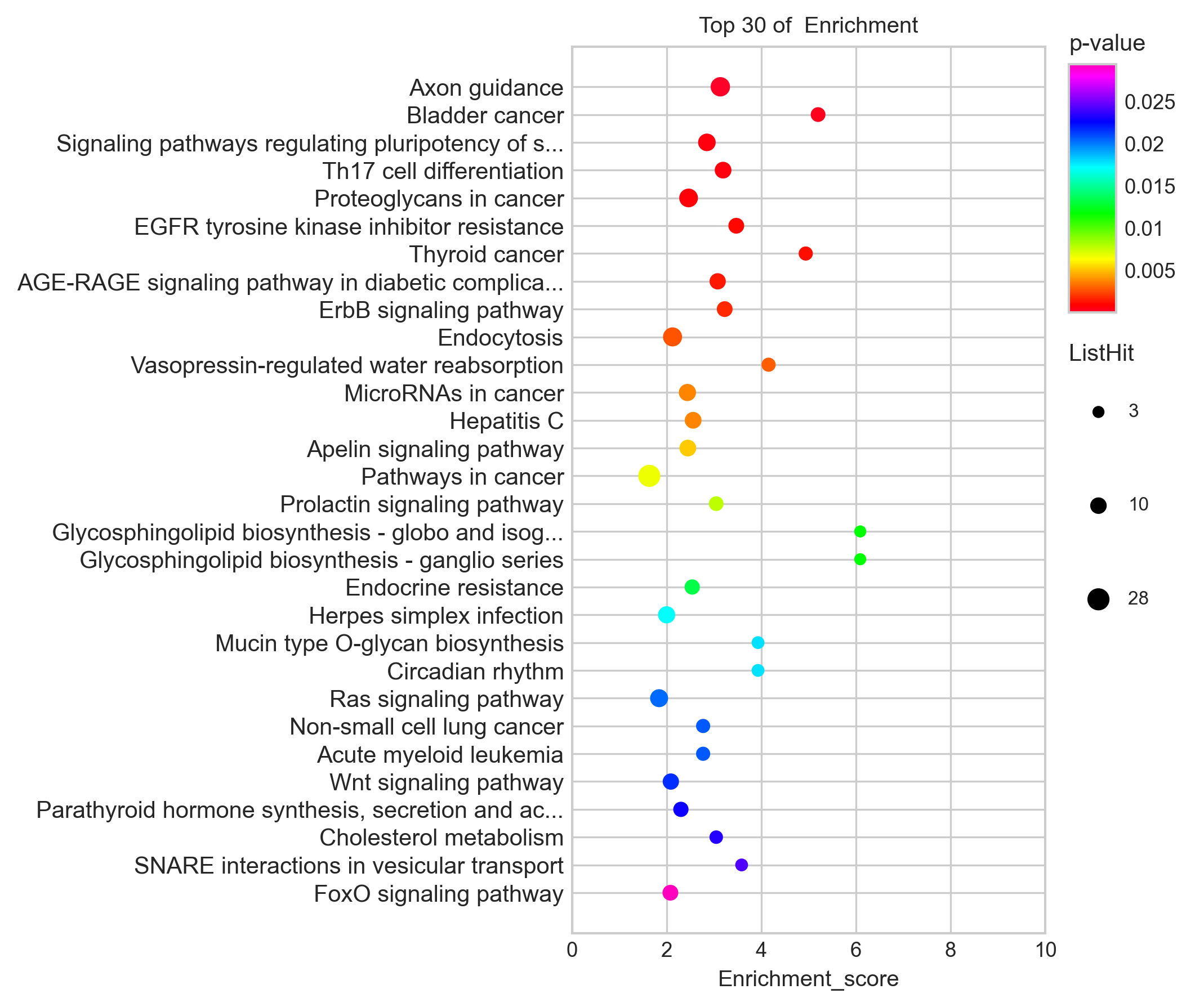

Supplement: Supplementary file 1 — Additional file 1. KEGG enrichment analysis of top 30 miRNAs. [file 12967_2022_3412_MOESM1_ESM.png]
